# Polish Adaptation and Validation of The Physical Appearance Comparison Scale (PACS) - an Analysis Among Young People in Late Adolescence in The Context of Obesity

**DOI:** 10.34763/devperiodmed.20172103.213223

**Published:** 2017-10-28

**Authors:** Anna Dzielska, Joanna Mazur, Hanna Nałęcz, Anna Oblacińska, Małgorzata Strucińska

**Affiliations:** 1 Departament of Child and Adolescent Health Warsaw, Poland; 2 Departament of Nutrition Institute of Mother and Child, Warsaw, Poland

**Keywords:** PACS, obesity, eating behaviours, body image, skala PACS, otyłość, zachowania żywieniowe, obraz ciała

## Abstract

**Introduction:**

The PACS scale is a tool which is widely used in foreign studies to evaluate the tendency towards appearance comparisons in social situations. People inclined to make such comparisons reveal a higher level of dissatisfaction with their body and a higher inclination towards problem eating.

**Purpose of the study:**

The main purpose of the study was to adapt the PACS scale. A factor structure assessment and reliability analysis of the Polish version was carried out. The correlation between PACS and pubertal development indicators, the body mass index and psycho-social factors and eating behaviours were evaluated as part of the validity analysis.

**Material and methods:**

The data were derived from the Internet-based study of problem eating behaviours conducted by the Institute of Mother and Child during the 2014/2015 school year. The analyses covered 1285 second grade upper secondary school students (47.2% boys). The mean age was 17.59 years (SD=0.39). An exploratory and confirmatory factor analysis of the PACS questionnaire was performed. Using the Kruskal-Wallis test or the Pearson’s r correlation, the association between (PACS-PL) and perceived pubertal timing, BMI, the body image (BIS), self-perception of body mass, self-esteem (RSES), self-esteem in social relations (SPPA-SSE), problem eating behaviours (TFEQ-13) were evaluated. The linear regression method was used to estimate the impact of PACS-PL on selected variables in the BMI groups in order to investigate of the moderation effect.

**Results:**

The shortened 3-item Polish version of the scale (PACS-PL) was considered optimal. It is characterized by high reliability (Cronbach’s α=0.868), and the main factor explains 79.1% of the variance of the scale results. The model also shows high values of fit indicators: χ2 = 1.144 (df=1, p=0.285), GFI=0.999, AGFI=0.996, CFI= 1.000, NFI=0.999, TLI- 1.000, RMSEA=0.011. Girls display a stronger tendency to compare their appearance with others. The PACS-PL scale demonstrates the expected correlations with developmental, psycho-social and behavioural factors, this correlation being stronger among girls. It was also shown that the PACS-PL index is a stronger predictor of dissatisfaction with the body and lower self-esteem in the group of obese adolescents.

**Conclusions:**

The Polish version of the scale, PACS-PL, is a reliable and valid tool for evaluating the tendency to compare one’s appearance with others in late adolescence. The subject of social comparison ought to become an element of prevention activities associated with acceptance of one’s body and self-esteem, especially among young people with excess body weight.

## Streszczenie

**Wstęp:**

Skala PACS jest szeroko stosowanym w badaniach zagranicznych narzędziem służącym do oceny tendencji do porównywania własnego wyglądu w sytuacjach społecznych. Osoby o skłonności do dokonywania takich porównań wykazują wyższy poziom niezadowolenia ze swojego ciała i większą skłonność do problemowego jedzenia.

**Cel pracy:**

Głównym celem pracy była adaptacja skali PACS. Dokonano oceny struktury czynnikowej i rzetelności jej polskiej wersji. W ramach oceny trafności oceniono związek PACS ze wskaźnikami rozwoju płciowego, wskaźnikiem masy ciała (BMI), czynnikami psychospołecznymi i zachowaniami związanymi z jedzeniem.

**Materiał i metody:**

Dane pochodziły z internetowego badania problemowych zachowań żywieniowych prowadzonego przez Instytut Matki i Dziecka w roku szkolnym 2014/2015. Analizy obejmują 1285 uczniów II klas szkół ponadgimnazjalnych (47,2% chłopców), w średnim wieku 17,59 lat (SD=0,39). Przeprowadzono eksploracyjną i konfirmacyjną analizę czynnikową kwestionariusza PACS. Za pomocą testu Kruskala-Wallisa lub korelacji r Pearsona oceniono związek PACS z postrzeganym tempem dojrzewania płciowego, BMI, obrazem ciała (BIS), samooceną masy ciała, poczuciem własnej wartości (RSES), poczuciem własnej wartości w relacjach społecznych (SPPA-SSE), problemowym sposobem jedzenia (TFEQ-13). Metodą regresji liniowej oszacowano wpływ PACS na wybrane zmienne w grupach BMI, jako badanie efektu moderacji.

**Wyniki:**

Za optymalną uznano skróconą 3-itemową polską wersję skali (PACS-PL). Posiada ona wysoką rzetelność (alfa-Cronbacha=0,868), a główny czynnik wyjaśnia 79,1% wariancji wyników skali. Model prezentuje wysokie wartości wskaźników dopasowania: χ 2 = 1,144 (df=1, p=0.285), GFI=0,999, AGFI=0,996, CFI=1,000, NFI=0,999, TLI-1,000, RMSEA=0,011. Dziewczęta prezentują silniejszą tendencję do porównywania wyglądu z innymi. Skala PACS-PL wykazuje spodziewane relacje z czynnikami rozwoju, psychospołecznymi i behawioralnymi, a związek jest silniejszy wśród dziewcząt. Wykazano, że indeks PACS-PL jest silniejszym predyktorem niezadowolenia z ciała i niższego poczucia własnej wartości w grupie otyłych nastolatków.

**Wnioski:**

Polska wersja kwestionariusza PACS-PL jest wiarygodnym i trafnym narzędziem do oceny tendencji do porównywania wyglądu z innymi w grupie nastolatków. Tematyka porównań społecznych wyglądu powinna stanowić element działań profilaktycznych związanych z akceptacją ciała i poczuciem własnej wartości, zwłaszcza wśród nastolatków z nadmiarem masy ciała.

## Introduction

According to L. Festinger’s Theory of Social Comparisons [1], comparing oneself with others is one of the methods of acquiring knowledge about oneself [2] and self-evaluation [3]. Study results reveal a connection between the tendency to compare one’s appearance with others, self-esteem [4], a distorted image of one’s own body and eating disturbances [5]. The consequences of the comparison process depend on the direction of comparison (“upwards” or “downwards”) [6], or on the characteristic features of the object of comparison, affecting the way one perceives oneself and one’s self-esteem. Social comparison may have a universalistic or particularistic nature [7]. The former comprises a wide social context and refers to society in general, the influence of the media and culture-forming factors. The latter is a comparison with people from one’s surroundings who are close, or similar to us, or with whom we identify. Comparisons with others having similar features affect the stability and adequacy of self-evaluation to a greater extent than comparisons with unknown persons [8]. In the context of appearance, particularistic comparisons cause more fear and anxiety associated with the body than a reference to universalistic standards [9]. Comparing oneself with someone whom we perceive as inferior, e.g. fatter, having an inferior appearance, i.e.“downwards” comparison, results in positive reinforcement leading to increasing self-esteem and satisfaction with one’s body. A comparison with “superior”, more attractive people, or “upwards” comparison, contributes to greater self-criticism, diminished self-esteem, dissatisfaction with one’s body and eating problems [1, 10]. Three types of motives which lead to making such comparisons are also worth noting, as they are associated with the direction of the comparisons. Apart from the need of self-evaluation, they may also include intended self-improvement and self-enhancement [3, 8].

From the point of view of body image, adolescence is a critical period for its proper development. As part of the development process, the image of the body is subject to gradual changes caused by puberty. These changes take place side by side with changes in physical appearance [11]. The speed of such change and its concurrence with peer development plays a significant role in accepting it and an adequate perception of one’s body.

Pubertal development, particularly if it occurs earlier than among peers, is associated with a negative image of the body among girls and on the contrary, a more positive approach to one’s body among boys [11, 12]. Berger et al. showed that girls who mature earlier or later than their peers are more likely to experience eating disorders [13].

The role of appearance in making social comparisons is widely described in studies using the approach known as the *Tripartite Influence Model* [14]. According to the above theory, comparing one’s appearance with others, apart from internalizing ideal beauty standards, plays the role of a mediator in the relations between the influence of peers, parents and the media [15], and dissatisfaction with one’s body [16] or body weight. Consequently, it predicts an increase in the drive to thinness [17] leading to disturbed eating [18].

The PACS scale is the most frequently used one to measure in studies of appearance self-evaluation based on social comparisons. There are several versions of the scale, which contain five to forty items. The scale was adopted in many countries and in various age and gender groups. The 11-item version of the scale (PACS-R) was used in American studies among women conducted by L.M. Schaefer [19], the same one was applied in German research by S. Claire Mölbert et al.[20]. It was also adjusted for the study of Iranian female undergraduate students by M. Atari et al. [21]. A tool comprising 5 questions was validated among French undergraduate students (over 80% women) and reduced to a 4 item version [22].

To the best of our knowledge there are no studies in Polish research concerning the area of comparing one’s appearance with others and no Polish tool exists to measure the phenomenon. Thus, an adaptation and validation of the tool tested on a group of older adolescents was performed, alongside with investigations for its possible correlation with the body mass index (BMI).

## Aim of the study

The main purpose of the study was to obtain a Polish adaptation of the *Physical Appearance Comparison Scale* (PACS). The adaptation evaluated the factor structure and reliability of the Polish version of the questionnaire. The distribution of the scale in the Polish youth population in late adolescence was also studied, taking into account the gender of the respondents. The construct and convergent validity evaluation also assessed the correlation between PACS and indicators of pubertal development, the body mass index (BMI), psycho-social factors and eating behaviours.

In line with the title of the study additional analyses in the context of the determinants and effects of obesity were planned. Further analyses with a stratification into body weight categories were performed. In addition it was verified whether the BMI is a moderator of the correlations between PACS and selected psychological variables.

### The persons studied

The data were derived from the study of problem eating behaviours (acronym SZAMKA) performed during the school year 2014/2015 by the Institute of Mother and Child 1Internal project performed in the Institute of Mother and Child in 2014-2015 entitled: “Problem eating behaviours (PEB) and their correlation with changes in the body mass index (BMI) in the period from late adolescence to early adulthood. Prospective study”. ^2^Opinion of the Bioethical Committee of the Institute of Mother and Child in Warsaw, No. 29/2014 of 17.11.2014. on a nationwide sample of 1300 upper secondary school students. Out of the entire sample of 1300 students the data of 1285 adolescents were selected (47.2% boys) after eliminating the missing data in all items of the tested PACS questionnaire. The average age of the respondents was 17.59 (SD=0.39), within the range of 15.8-19.8 years.

The study was performed by the survey using the internet-assisted method (CAWI – Computer Aided Web Interviewing). The young people completed the questionnaire in the school computer laboratory or at home after obtaining a link to the temporary study website, www.szamka.org All the participants signed the document of informed consent. The study procedure, the applied research tool and the consent form were approved by the Bioethical Committee of the Institute of Mother and Child^2^.

## Methods

### PACS (Physical Appearance Comparison Scale)

An original tool for evaluating one’s appearance in comparison with others composed of 5 questions created by J.K. Thompson et al. was tested [14]. The tool was subjected to translation and back-translation: the Polish language version, reviewed by a team of specialists, was then translated back into English and compared with the original tool. after introducing the necessary corrections in the Polish version, the scale was tested in a pilot study on a sample of 128 second grade upper secondary school students. No objections to the wording of the individual items were raised in the pilot study, which led to using the same version in the main SZAMKA survey. Table I shows the original language version of the scale and its final translation into Polish.

**Table I:** English language version of PACS with a translation into Polish. Tabela I. Angielska wersja językowa skali PACS wraz z tłumaczeniem na język polski.

	PACS – English version	PACS –Polish version*
	Using the following scale please select a number that comes closest to how you feel:	Zakreśl odpowiedź na poniższe stwierdzenia, która najbardziej opisuje co czujesz.
	0 Never; 1 Seldom; 2 Sometimes; 3 Often; 4 Always	0 Nigdy; 1 Rzadko; 2 Czasami; 3 Często; 4 Zawsze
PACS_1	1. At parties or other social events, I compare my physical appearance to the physical appearance of others.	1. Na imprezach lub innych spotkaniach towarzyskich porównuję mój wygląd z wyglądem innych osób.
PACS_2	2. The best way for a person to know if they are overweight or underweight is to compare their figure to the figure of others.	2. Najlepszy sposób, by dowiedzieć się, czy ma się nadwagę lub niedowagę, to porównanie własnej sylwetki z sylwetkami innych osób.
PACS_3	3. At parties or other social events, I compare how I am dressed to how other people are dressed.	3. Na imprezach lub innych spotkaniach towarzyskich porównuję mój ubiór z ubiorem innych osób.
PACS_4	4. Comparing your „looks” to the „looks” of others is a bad way to determine if you are attractive or unattractive.	4. Porównanie swojego wyglądu do wyglądu innych osób to zły sposób na określenie własnej atrakcyjności lub nieatrakcyjności.
PACS_5	5. In social situations, I sometimes compare my figure to the figures of other people.	5. W sytuacjach towarzyskich czasem porównuję moją sylwetkę z sylwetkami innych osób.

* Polish version of the PACS scale (PACS-PL) recommended in this study consists of 3 items: PACS_1, PACS_3, PACS_5. ^*^ Polska wersja skali (PACS-PL) rekomendowana w tym badaniu składa się z następujących stwierdzeń: PACS_1, PACS_3, PACS_5.

The original scale was composed of five statements with a choice of five categories of answers scoring 0 to 4 points. The summary index of the 5-item scale ranges from 0 to 20. The Polish version of the scale recommended in the study consisted of 3 statements and its summary index ranged from 0 to 12 points. A higher score indicates a greater tendency to compare one’s appearance with others in social situations.

### Body image

For the purpose of evaluating body image the *Body Image Subscale* (BIS) was used, which is an element of a tool used for examining the experience with one’s body (Body Investment Scale) developed by I. Orbach and M. Mikulincer [23]. The BIS scale is composed of six items with five responses, from *definitely disagree to definitely agree*. Each answer scored 0 – 4 points, which gives a summary score from 0 to 24 points. A higher score indicates a more positive approach to one’s body.

### Body weight self-perception

The young people were asked to answer the question: *Do you think your body is….: much too thin, a a bit too thin, about the right size, a bit too fat, much too fat*. Their perceived body size was examined in this way, so as to identify those participants who are dissatisfied with their body weight. In the form presented, the question has been included in HBSC protocols since 2001/02 and was tested numerous times in population studies [24]. During the analyses the question was treated as a quasi-continuous scale.

### Self-esteem

*The Rosenberg Self−Esteem Scale* (RSES) developed for investigating global self-esteem and self-acceptance among youth was used to evaluate the self-esteem of young people [25]. In our study we used the Polish adaptation developed by K. Doroszewicz. It was employed in student studies entitled *The self-knowledge scale* and obtained good psychometric features [26]. The scale consists of 10 statements about the respondent with four categories of answers, scoring 0 to 3 points. The summary index of the scale ranges between 0 and 30. A higher score indicates a higher level of self-esteem.

### Sense of coherence

*The Sense of Coherence Scale for Adolescents* (SOC-11M) was used, the version being adapted for Polish studies by M. Zwoliński et al. [27]. Young people verified 11 statements which enabled a determination of their sense of coherence, resourcefulness and rationality in everyday life (e.g. *interest in what goes on around them, feeling that one is unfairly treated, understanding one’s thoughts and feelings* etc.). The coherence indicator was calculated by adding up the scores for each question. The scale ranged from 0 to 33 points, a high score indicated a higher level of coherence.

### Self-perception profile for adolescents – Social self-esteem

The *Social acceptance scale*, one of the modules included in the seven-item scale proposed by *S. Harter* was used [28]. The scale was adapted in the HBSC 2006 study. It enabled a description of the self-perception profile by adolescents. The module used in our study comprised 5 statements which concerned functioning in a peer environment. The answers scored from 0 to 3. The scale summary index ranged from 0 to 12 points, a higher score indicated a higher level of self-esteem in social relations.

### Eating behaviours

The *Three-Factor Eating Questionnaire* (TFEQ-13) questionnaire as adapted by the HBSC 2010 studies was used to evaluate problematic eating behaviours [29]. The questions of the TFEQ-13 scale consist of three dimensions:

- Cognitive Restraint of Eating– 5 questions; scale ranges from 0 to 15.
- Uncontrolled Eating – 5 questions; scale ranges from 0 to 15.
- Emotional Eating – 3 questions; scale ranges from 0 to 9.

The TFEQ-13 questionnaire contains answers from *definitely yes* (3 points) to *definitely no* (0 points). The last question of the questionnaire described on an eight-degree scale (1-8) referred to the assessment of the ability to restrict eating and was re-encoded in a 4-point scale (0-3). The values were calculated separately for each sub-scale. A high overall score in the partial scale indicated serious problems in that area.

### Perceived pubertal timing

The young people answered the question: *Do you think that your physical development and sexual maturation occurs(ed) earlier or later in comparison with most girls (boys) of your age*? There were five categories of answers: *much earlier, a bit earlier, similarly as in most girls (boys) of my age, a bit later, much later*. The question was re-encoded into 3 categories by combining extreme answers: *early starters, normal, late starters*.

### Body mass index

On the basis of the data obtained from young people their body mass index was calculated (BMI = body mass in kg/body height in cm^2^). The classification into BMI categories was conducted on the basis of the International Obesity Task Force (IOTF) criteria [30]. Four categories of body mass were distinguished: underweight, normal body weight, overweight and obesity. The BMI variable was analyzed as continuous and also divided into the above four categories. The mean BMI was 21.5 (SD=3,27).

## Methods of data analysis

Statistical analyses were conducted using the IBM SPSS v. 21 and AMOS v. 21 software. The following statistical methods were used:

- The α-Cronbach’s test to evaluate the reliability of PACS,
- In order to evaluate the factor structure of the scale exploratory factor, an analysis was performed using the principal component analysis taking into account Kaiser’s criterion and confirmatory factor analysis (CFA) were used. The rationale for performing the analyses was estimated on the basis of the applicability of factor analysis: the (KMO) (Kaiser-Meyer-Olkin Measure of Sampling Adequacy) and Bartlett’s Test of Sphericity. The value of the parameters was estimated using the maximum likelihood method and model fit indicators: CFI (comparative fit index), GFI (goodness-of-fit index), AGFI (adjusted goodness-of-fit index), NFI (normed fit index), TLI (Tucker-Lewis index), and RMSEA (root mean square error of approximation).
- In order to evaluate the normality of the distribution of all the variables, a Kolmogorov-Smirnov test was carried out, the value of skewness and kurtosis was established, as well as the size of the floor and ceiling effect.
- To evaluate the differences dependent on gender the non-parametric U Mann-Whitney test was used.
- In order to evaluate the validity of the tool the association between the PACS-PL and the following variables were investigated:
  º Perceived pubertal timing, by comparing the average PACS-PL index in three pubertal groups - Kruskall-Wallis test;
  º Psycho-social factors (evaluation of one’s own body image, self-perception of body weight, self-esteem, sense of coherence, self-esteem in social relations), behavioural factors (applying diet restrictions, emotional eating, loss of control over eating) - Pearson’s correlation with PACS-PL.
- Using the full matrix of correlation between PACS-PL, BMI and the above psycho-social factors selected, a direct and indirect relationship relating to obesity determinants was indicated.
- In order to verify whether BMI is a moderator of selected correlations between PACS and psycho-social variables, univariate linear regression models specific for the body weight category were estimated. In these models (estimated by the enter method) PACS-PL was an independent variable, while one’s own body image or self-esteem were dependent variables.

## Results

### Evaluation of the factor structure and reliability of PACS

In the first step of the analyses the **5-item version of the scale** was subject to exploratory factor analysis using the principle component analysis, without a specific number of distinguished factors. The analysis indicated that the scale consisting of five items creates a univariate structure. The values of the factor loadings of the 4 statements ranged from 0.695 to 0.869. The factor loading of statement No. 4 assumed a very low value – 0.259. Moreover, confirmatory factor analysis showed that the 5-item model of PACS was not well fitted to the data (tab. III). The 5-item model explained 56.15% of the total variability of the scale.

An analysis of the distribution of all PACS items indicated significant deviation from normality. Four items were positively skewed and one was negatively skewed. The floor effect ranged from 16.5% to 45.5% (question No. 2), and the ceiling effect was from 1.8% (question No. 2) to 21.8% (tab. II).

Due to the inadmissible value of the factor loading of one of the statements as a next step, an exploratory factor analysis of **a 4-item scale** was carried out, excluding question No. 4. The shortened version of the scale omitting the same question was also tested in French and American studies.

The scale thus constructed formed a univariate structure and the factor loadings of successive questions assumed values in accordance with the recommended statistical criteria (from 0.708 to 0.879). The 4-item model explained 69.09% of the total scale variability. The reliability coefficient (α-Cronbach’s) for the shortened, 4-item scale amounted to 0.851.

As a further step a confirmatory factor analysis of the above scale was performed. Due to the indicators obtained, which revealed the 4-item model to be unfit, a decision was made to remove yet another statement from the scale (question No.2), which according to previous analyses revealed a relatively high floor effect (45.5%).

**Fig. 1:**
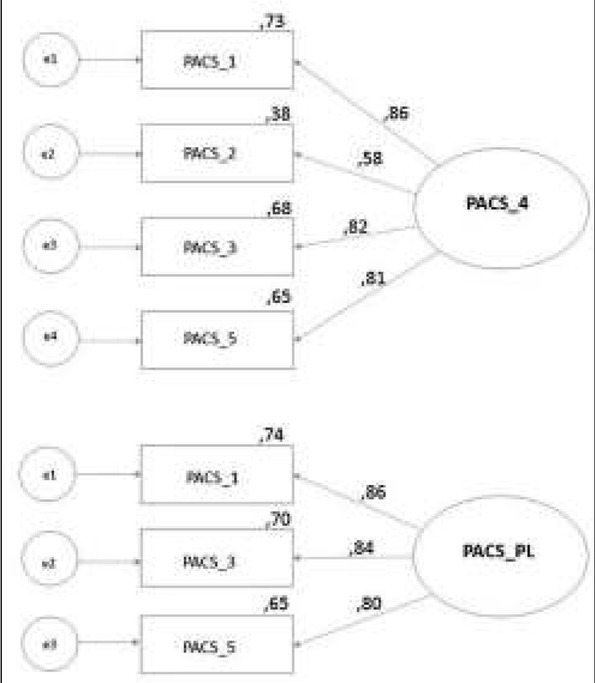
Confirmatory Factor Analysis of 4-item (PACS_4) and 3-item (PACS-PL) models – standardized regression weights and squared multiple correlations. Ryc. 1. Konfirmacyjna analiza czynnikowa 4-itemowej (PACS_4) i 3-itemowej (PACS_PL) skali PACS – standaryzowane współczynniki regresji i współczynniki r2.

The parameters of the **3-item model** show that the model was well fitted and factor loadings of all the questionnaire items were appropriate, which made it acceptable. The value of fit indices of the models tested (5, 4 and 3 items) are presented in Table 3. The diagrams display the values of standardized regression weights and squared multiple correlations (fig. 1).

**Table II:** Skewness and kurtosis values, floor and ceiling effect and significance level of the Kolmogorov-Smirnov (K-S) test of all PACS items. Tabela II. Wartość skośności i kurtozy, efektu podłogi i sufitu oraz poziom istotności testu Kołmogorova-Smirnova (K-S) dla składowych skali PACS.

Item numer *Numer pytania*	Skewness *Skośność*	Kurtosis *Kurtoza*	p*	Floor effect (%) *Efekt podłogi (%)*	Ceiling effect (%) *Efekt sufitu (%)*
PACS_1	0.496	-0.699	<0.001	28.8	6.0
PACS_2	0.895	-0.113	<0.001	45.4	1.8
PACS_3	0.329	-0.820	<0.001	25.7	6.1
PACS_4	-0.091	-1.186	<0.001	16.5	21.8
PACS_5	0.444	-0.775	<0.001	29.3	6.5

* On the basis of K-S test. ^*^ Na podstawie testu K-S.

**Table III:** Comparison of 4 and 3-item structural model parameters. Tabela III. Porównanie parametrów modelu 4- i 3-itemowego.

	Goodness-of-fit measure/*Miary dopasowania*							
	Model χ2 *Ogólny test χ2*		Absolute fit *Bezwględne dopasowanie*		Incremental fit *Przyrostowe dopasowanie*		Error of approximation *Błąd aproksymacji*	
	CMIN	P	GFI	AGFI	NFI	TLI	RMSEA	90%CI
5-item model Model 5-itemowy	18.280 d.f.=5	<0.001	0.987	0.960	0.981	0.966	0.080	0.060-0.102
4-item model Model 4-itemowy	18.280 d.f.=2	<0.001	0.993	0.964	0.992	0.979	0.080	0.049-0.115
Model 3-item 3-model itemowy	1.144 d.f.=1	0.285	0.999	0.996	0.999	1.000	0.011	0.000-0.076

The Polish version of the scale (PACS-PL) used to evaluate the degree of comparison of one’s appearance with others, shortened as a result of the analyses, consists of 3 items (questions 1, 3 and 5 of the original scale) and has a univariate structure. The value of the KMO index is 0.736, and Bartlett’s sphericity test is statistically significant (p<0.001). All the items in the scale are significantly correlated. The size of the total variation explained was 79.1% and reliability was at α-Cronbach’s=0.868 level.

### Distribution of the PACS-PL scale in the population of older adolescents

The summary, 3-item PACS-PL scale ranges from 0 to 12 points. A high score indicates a strong tendency to compare one’s appearance with others. The scale is not normally distributed, showing a relatively small positive asymmetry (SKE=0.411, SE_SKE_=0.068) and negative kurtosis in comparison to the normal distribution (K=-0.551, SE_K_=0.136).

The average PACS-PL index was 4.29 (SD=3.18). An analysis by the U Mann-Whitney rank test indicated that it is significantly higher in the group of girls (M=5.23, SD=3.15) than in boys (M=3.27, SD=2,88), U= 131.87; p<0.001.

### Association between the evaluation of one’s appearance in comparison with others (PACS-PL) and perceived pubertal development

The mean PACS-PL index was higher among girls who underwent puberty later or earlier than in the group of their friends who matured at approximately the same time as their peers (p<0.001). The pubertal timing in boys did not reveal a relationship with PACS-PL (tab. IV).

### Correlation between PACS-PL and psycho-social and behavioural factors and BMI

The correlation between PACS-PL and psycho-social and behavioural factors and the BMI was investigated. On the basis of the analysis conducted on a full sample of young people, Table V presents the values of correlation ratios between variables including significance levels (p). PACS-PL showed significant correlations with all the indicators. In line with expectations a negative correlation was recorded between PACS-PL and the body image (BIS), sense of coherence (SOC-11M), self-esteem (RSES) and self-esteem in social relations (SPPA-SSE). PACS-PL also had a negative correlation with the BMI, although the value of the r-Pearson’s ratio was very low (r=-0.13). The analyses conducted separately in the group of boys and girls showed the absence of significant correlation between these variables. Self-perception of body weight had a positive correlation with PACS-PL (only among girls), as well as all dimensions of the TFEQ-13 questionnaire (for both genders). Stronger correlations were observed in the girls’ group. The value of r-Pearson’s ratios were higher in the group of girls than boys with the exception of the relationship between PACS-PL and self-esteem (scale 4), where the values of the ratios were similar for both genders.

### The BMI category as a moderator of correlations between appearance social comparison and satisfaction with one’s body and self-esteem

Univariate linear regression models specific for the BMI categories (underweight, norm, overweight, obesity) were estimated in order to determine the impact of appearance in social comparisons (PACS-PL) on body image (BIS) and self-esteem (RSSE) (tab. VI). A higher score on the PASC-PL scale was associated with significant deterioration of body image in all the groups distinguished. However, in the group of obese teenagers, comparison of appearance explained the highest variability of the BIS index (R^2^=21%). PASC-PL also proved to be a negative predictor of self-esteem in groups of adolescents with normal body weight, overweight and obese persons. The highest percentage of RSES variation explained by PACS-PL was also recorded in the obese group (R^2^=23%).

## Discussion

The main purpose of the study was to adapt the Polish version of the PASC questionnaire which examines the tendency to compare one’s appearance with others. As a result of the analysis conducted in late adolescence, a 3-item version of the scale (PACS-PL) was obtained, with good psychometric qualities and high reliability. The validity of the questionnaire we developed was confirmed by a significant positive correlation with other body image assessment tools validated earlier among Polish adolescents. When discussing the results obtained, the issues defined in the purpose of the study must first be taken into account: the factor structure of the Polish version of the scale (PACS-PL), differences associated with gender and the usefulness of the tool adapted in studies taking into account the determinants and effects of obesity.

**Table IV:** Mean PACS-PL index in groups depending on perceived pubertal timing, total and by gender. Tabela IV. Średni indeks PACS-PL w zależności od postrzeganego tempa dojrzewania płciowego, ogółem i według płci.

Perceived pubertal timing *Postrzegane tempo dojrzewania*	Total *Ogółem*			Boys *Chłopcy*	Girls *Dziewczęta*
	N	%	M±SD	M±SD	M±SD
*Wcześniej* Early starters *dojrzewający*	311	24.2	4.27±3.32	3.08±2.85	5.69±3.28
*Przeciętnie* Normal *dojrzewający*	832	64.7	4.26±3.09	3.33±2.86	4.98±3.08
Late starters *Później dojrzewający*	142	11.1	4.87±3.66	3.45±3.30	6.46±3.39
χ2			=2.687	=0.844	=13.786
p*			=0.261	=0.656	<0.001

* On the basis of the Kruskal Wallis test. ^*^ Na podstawie testu Kruskala Wallis’a.

**Table V:** Descriptive statistics and correlation matrix for study variables, total sample. Tabela V. Statystyki opisowe i macierz korelacji dla analizowanych zmiennych, ogółem.

		1	2	3	4	5	6	7	8	9
(M SD)		(4.33 3.22)	14.57 (5.27)	14.57 (5.27)	(2.23 0.96)	22.49 (8.42)	7,68 (2,01)	6,23 (3,59)	2,74 (2,32)	5,91 (3,26)
1	PACS-PL	1								
2	BIS	-.329^***^	1							
3	BWS	.233^***^	-.340^***^	1						
4	RSES	-.231^***^	.554^***^	-.127^***^	1					
5	SOC	-.276^***^	.401^***^	-.060^*^	.479^***^	1				
6	SPPA-SSE	-.089^**^	-.092^**^	-.032	-.163^***^	-.141^***^	1			
7	TFEQ-RE	.179^***^	-.299^***^	.375^***^	-.129^***^	-.174^***^	.085^**^	1		
8	TFEQ-EE	.208^***^	-.321^***^	.098^***^	-.336^***^	-.383^***^	.094^**^	.206^***^	1	
9	TFEQ-UE	.149^***^	-.156^***^	-.119^***^	-.155^***^	-.334^***^	.078^**^	.027	.574^***^	1
10	BMI	-.126^***^	-.093^**^	.340^***^	.047	.013	.002	.203^***^	.028	-.041

* p<0,05; ^**^ p<0,01; ^***^p<0,001 Abbreviations: 1 Physical Appearance Comparison scale – Polish Version; 2 Body Image Scale; 3 Body weight self-perception; 4 Rosenberg Self-Esteem Scale; 5 Sense of Coherence; 6 Self-Perception Profile for Adolescents – Social Self-Esteem; 7 Three Factor Eating Questionnaire – Restrictive Eating; 8 Three Factor Eating Questionnaire – Emotional Eating; 9 Three Factor Eating Questionnaire – Uncontrolled Eating *Wykaz skrótów*: 1 Skala oceny wyglądu w porównaniu z innymi- wersja polska; 2 Skala Obrazu Własnego Ciała; 3 Samoocena masy ciała; Poczucie własnej wartości wg Rosenberg; 5 Poczucie koherencji; 6 Profil samooceny dla nastolatków – poczucie własnej wartości w relacjach społecznych; 7 Kwestionariusz Zachowań Związanych z Jedzeniem – Stosowanie restrykcji dietetycznych; 8 Kwestionariusz Zachowań Związanych z Jedzeniem – Jedzenie pod wpływem emocji; 9 Kwestionariusz Zachowań Związanych z Jedzeniem – Utrata kontroli nad jedzeniem;

**Table VI:** Linear regression model summaries with PACS-PL as predictor. Tabela VI. Podsumowanie modeli regresji liniowej ze skalą PACS-PL jako predykotrem.

Dependent variable *Zmienna zależna*	Underweight *Niedobór masy ciała*		Normal *Norma*		Overweight *Nadwaga*		Obesity *Otyłość*	
	N	64	N	1035	N	126	N	60
	%	5,0	%	80,5	%	9,8	%	4,7
	(β CI)	p	(β CI)	P	(β CI)	p	(β CI)	p
BIS	-0.355 (-0.820- -0.162)	<0.01	-0.325 (-0.623- -0.435)	<0.001	-0.344 (-0.792 - -0.274)	<0.001	-0.458 (-1.415- -0.460)	0<0.001
R2	=0.126		=0.105		=0.118		=0.210	
RSES	-0.086 (-0.505- 0.248)	=0.497	-0.232 (-0.499- -0.296)	<0.001	-0.180 (-0.608- -0.009)	<0.05	-0.478 (-1.269- -0.443)	<0.001
R2	=0.007		=0.053		=0.025		=0.229	

### Factor structure of the PACS scale

In the course of the analyses two questions were removed from the original version of the tool (number four (PACS_4) and subsequently number two (PACS_2)). The shortened 4-item version of the PACS scale was also used in French and American studies. The reason for omitting question 4 was its low correlation with other components of the scale (squared multiple correlation <0.05) [31] or low internal correlation factor (<0,2) [22]. Both the foreign versions of the 4-item scale were characterized by good reliability, as was the model tested in this study. The main factor explained the 56% variance in the Polish sample, which is similar to the results obtained in the abovementioned studies by French and American authors. In the Polish version of the 4-item scale attention was paid to question No. 2 (PACS_2), which showed unacceptable skewness of distribution. Similarly, the results of the confirmation factor analysis unequivocally indicated that the 4-item model was unsuitable and the best one is a 3-item model, obtained after omitting question number 2 (PACS_2). In the future however, the 4-item model is also worth taking into account and a comparison of the parameters of both models in other populations ought to be conducted.

### Differences of appearance evaluation associated with gender

It was found that girls show a much higher tendency to make social comparisons of their appearance than boys. According to S. Shon, among other factors the differences associated with gender may result from a different approach to the body by boys and girls [32]. Girls are more often dissatisfied with their body and more likely than boys to experience body image concerns, which is expressed e.g. by an inadequate assessment of their weight [33]. The appearance of girls and young women is more often the object of interest for the media and peer conversations than the appearance of boys [33]. Girls are more likely to experience negative comments associated with their appearance than boys, and are more often under pressure to look according to the expected standards [34]. Another important factor is the type of social comparison dependent on gender and the motives which accompany it. Girls mainly compare their appearance with others [35], and their purpose is to assess themselves. [32]. Boys usually make social comparisons in order to evaluate their abilities against the background of other persons (e.g. in sport), and the result of the comparison is inspiring for them and motivates them to improve their skills or is a source of self-enhancement [35]. Greater concentration on their body among girls is expressed by an increased tendency to seek feedback about their appearance and attractiveness by comparison with other persons. Another result of this study which confirms this thesis is the connection between appearance social comparisons (PACS-PL) and the level of puberty observed among girls. Puberty that is too early or delayed (i.e. being temporarily “different” from others) tends to increase the tendency to the self-evaluation of one’s appearance on the basis of observations of other people in one’s surroundings.

It was demonstrated that there is a connection of PACS-PL with psycho-social and behavioural factors, which on the one hand confirms the validity of the tool adapted and on the other sets the directions for future analyses. Intensified social comparisons of appearance among both genders are accompanied by a more negative approach to one’s own body, lower self-esteem, lower perception of social acceptability, lower sense of coherence, more frequent diet restrictions, increased tendency to emotional eating and loss of control over eating. In addition an increased tendency to make social comparisons is associated with a more negative assessment of own body weight among girls.

### PACS-PL results and body weight

The analyses did not confirm a direct relationship between the tendency to compare one’s appearance and body weight measured by the BMI. However, on the basis of the results obtained, it is to be concluded that comparing oneself with others may indirectly affect the obesity risk. PACS-PL significantly correlated with indicators associated with increases in BMI (applying diet restrictions, self-assessment of body weight). A similarly perceived level of puberty which affects PACS is also a recognized obesity risk factor [36].

In addition, after stratification into body weight categories, it was demonstrated that the body mass index is a moderator of the association between the tendency to compare one’s appearance in social situations and satisfaction with one’s body and self-esteem. Among obese adolescents the impact of PACS on the above dependent variables was stronger than among their peers who are overweight, normal or underweight.

The absence of a direct connection between the PACS scale results and the body mass index was also described in Australian studies concerning adolescents aged 12-16 years. By applying structural modeling Webb et al. showed that BMI and social comparisons are independent predictors of dissatisfaction with one’s appearance but do not correlate with each other. In addition, it was demonstrated that a social comparison of appearance is a mediator of the correlations between peer pressure to be attractive and dissatisfaction with one’s appearance. Conversely, dissatisfaction with one’s appearance directly affected the sense of social rejection, which was the main object of the study [37]. As such, social comparison of appearance may be treated as a factor which affects health and social functioning, for instance a sense of rejection, which, in the light of other studies, frequently applied to persons with above-normal body weight [38].

## Practical conclusions and implications

1. The 3-item Polish version of the PACS scale (PACS-PL) has good psychometric qualities and may be used for studies of adolescents. In the future, however, the extended 4-item model should also be taken into consideration.
2. Appearance comparisons do not reveal a direct connection with the body mass index. However, mutual indirect correlations ought to be noted.
3. When comparing one’s appearance with others among young people must be treated as a phenomenon having deep individual and cultural determinants. Due to the connection between PACS-PL and obesity risk factors (e.g. diet restrictions) knowledge about social comparison mechanisms ought to be used in multi-directional obesity prevention programs.
4. An issue which must be remembered when designing tailored prevention programs is the diagnosis of the target population from the point of view of the tendency to make social comparisons. In the group with a high tendency to make such comparisons special attention must be paid to reinforcing internal resources, such as self-esteem.

## References

[1] Festinger L (1954). A Theory of Social Comparison Processes. Human Relations.

[2] Buunk AP, Gibbons FX (2007). Social comparison: The end of a theory and the emergence of a field. Organ Behav Hum Decis Process.

[3] Corcoran K, Crusius J, Mussweiler T, Chadee D. (2011). Theories in social psychology.

[4] Tylka TL, Sabik NJ (2010). Integrating Social Comparison Theory and Self-Esteem within Objectification Theory to Predict Women’s Disordered Eating. Sex Roles.

[5] Pokrajac-Bulian A, Ambrosi-Randić N, Dobrila J, Kukić M (2008). Thin-Ideal Internalization and Comparison Process as Mediators of Social Influence and Psychological Functioning in the Development of Disturbed Eating Habits in Croatian College Females. Psychol Top.

[6] Wheeler L, Suls J, Wheeler L (2000). Handbook of Social Comparison: Theory and Research.

[7] Miller DT, Turnbull W, McFarland C (1988). Particularistic and universalistic evaluation in the social comparison process. J Pers Soc Psychol.

[8] Wood JV (1989). Theory and Research Concerning Social Comparisons of Personal Attributes. Psychol Bull.

[9] Heinberg LJ (1992). Thompson JK. The effects of figure size feedback (positive vs. negative) and target comparison group (particularistic vs. universalistic) on body image disturbance. Eating Disorders.

[10] Bailey SD, Ricciardelli LA (2010). Social comparisons, appearance related comments, contingent self-esteem and their relationships with body dissatisfaction and eating disturbance among women. Eating Behaviors.

[11] Markey ChN. (2010). Invited Commentary: Why Body Image is Important to Adolescent Development. J Youth Adolescence.

[12] Ge X, Elder C, Regnerus M, Cox C (2001). Pubertal transitions, perceptions of being overweight, and adolescents’ psychological maladjustment: Gender and ethnic differences. Soc Psychol Q.

[13] Berger U, Weitkamp K, Strauss B (2009). Weight limits, estimations of future BMI, subjective pubertal timing and physical appearance comparisons among adolescent girls as precursors of disturbed eating behaviour in a community sample. Eur Eat Disord Rev.

[14] (1991). Thompson JK, Heinberg L, Tantleff S. The Physical Appearance Comparison Scale (PACS). The Behavior Therapist.

[15] Fardouly J, Vartanian JR (2015). Negative comparisons about one’s appearance mediate the relationship between Facebook usage and body image concerns. Body Image.

[16] Rodgers RF, McLean SA, Paxton SJ (2015). Longitudinal Relationships Among Internalization of the Media Ideal, Peer Social Comparison, and Body Dissatisfaction: Implications for the Tripartite Influence Model. Dev Psychol.

[17] Ferreira C, Pinto-Gouveia J, Duarte C (2013). Physical appearance as a measure of social ranking: The role of a new scale to understand the relationship between weight and dieting. Clin Psychol Psychother.

[18] van den Berg P, Thompson JK, Obremski-Brandon K, Coovert M (2002). The Tripartite Influence model of body image and eating disturbance, A covariance structure modeling investigation testing the mediational role of appearance comparison. J Psychosom Res.

[19] Schaefer LM (2014). Thompson JK. The development and validation of the Physical Appearance Comparison Scale-Revised (PACS-R). Eat Behav.

[20] Claire Mölbert S, Hautzinger M, Karnath HO, Zipfel S, Giel K. (2017). [Validation of the Physical Appearance Comparison Scale (PACS) in a German Sample: Psychometric Properties and Association with Eating Behavior, Body Image and Self-Esteem]. Psychother Psychosom Med Psychol.

[21] Atari M, Akbari-Zardkhaneh S, Soufiabadi  M, Mohammadi L. (2015). Cross Cultural Adaptation of the Physical Appearance Comparison Scale Revised in Iran. Int J Body Mind Culture.

[22] Dany L, Urdapiletta I (2012). Validation of a French measure of body comparision: The Physical Appearance Comparision Scale. Revue international psychologie sociale.

[23] Orbach I, Mikulincer M (1998). Body Investment Scale. Construction and validation of body experience. Psychol Assess.

[24] Nemeth A, Ojala K (2005/2006). Body image and weight control behavior, HBSC Research Protocol for.

[25] Rosenberg M (1965). Society and the adolescent self-image.

[26] Forbes G, Doroszewicz K, Card K, Adams-Curtis L (2004). Association of the thin body ideal, ambivalent sexism and self−esteem with body acceptance and the preferred body size of college women in Poland and the United States. Sex Roles.

[27] Zwoliński M, Jelonkiewicz I, Kosińska-Dec K (2011). Skala poczucia koherencji dla młodzieży i jej właściwości psychometryczne. Sztuka leczenia.

[28] Harter S (1995). Manual for the Self-Perception Profile for Adolescents, University of Denver, Denver CO, 1998, [za] Wichstrom L. Harter’s Slef-Perception Profile for Adolescents: reliability, validity, and evaluation of question format. J Pers Assess.

[29] Dzielska A, Mazur J, Małkowska-Szultnik A, Kołoło H (2009). Adaptacja polskiej wersji kwestionariusza Three-Factor Eating Questionnaire (TFEQ-13) wśród młodzieży szkolnej w badaniach populacyjnych. Probl Hig Epidemiol.

[30] Cole TJ, Lobstein T (2012). Extended international (IOTF) body mass index cut-offs for thinness, overweight and obesity. Pediatr Obes.

[31] Davison TE, McCabe MP (2005). Relationships Between Men’s and Women’s Body Image and Their Psychological, Social, and Sexual Functioning. Sex Roles.

[32] Sohn SH (2010). Sex differences in social comparision motives in body image process. N Am J Psychol.

[33] Striegel-Moore RH, Franko DL, Cash TF, Pruzinsky T (2004). Body image. A handbook of theory, research, and clinical practice. The Guliford Press.

[34] Helfert S, Warschburger P (2013). The face of appearance-related social pressure: gender, age and body mass variations in peer and parental pressure during adolescence. Child Adolesc Psychiatry Ment Health.

[35] Tatangelo GL, Ricciardelli LA (2015). Children’s body image and social comparisons with peers and the media. J Health Psychol.

[36] Harris MA, Prior JC, Koehoorn M (2008). Age at menarche in the Canadian population: Secular trends and relationship to adulthood BMI. J Adolesc Health.

[37] Webb HJ, Zimmer-Gembeck MJ, Donovan CL (2014). The appearance culture between friends and adolescent appearance-based rejection sensitivity. Body Image.

[38] Park LA, Harwin MJ (2010). Visible versus non-visible rejection: consequences of appearance-based rejection sensitivity. J Res Pers.

